# Stability and prognostic value of Slug, Sox9 and Sox10 expression in breast cancers treated with neoadjuvant chemotherapy

**DOI:** 10.1186/2193-1801-2-695

**Published:** 2013-12-28

**Authors:** Cosima Riemenschnitter, Ivett Teleki, Verena Tischler, Wenjun Guo, Zsuzsanna Varga

**Affiliations:** Institute of Surgical Pathology, University Hospital Zurich, Zurich, Switzerland; 1st Department of Pathology & Experimental Cancer Research, Semmelweis University, Budapest, Hungary; Ruth L and Davis S Gottesman Institute for Stem Cell Biology and Regenerative Medicine, Department of Cell Biology, Albert Einstein College of Medicine, Bronx, NY USA; Institute of Surgical Pathology, University Hospital Zurich, Schmelzbergstrasse 12, CH 8091 Zurich, Switzerland

**Keywords:** Stem cells, Transcription factors, Stability, Chemotherapy, Prognosis, Breast cancer

## Abstract

**Background:**

Expression of transcription-factors as Slug and Sox9 was recently described to determine mammary stem-cell state. Sox10 was previously shown to be present also in breast cancer. Protein overexpression of Slug, Sox9 and Sox10 were associated with poor overall survival and with triple-negative phenotype in breast cancer. In this study we tested the stability of Slug, Sox9 and Sox10 expression during chemotherapy and addressed their prognostic role of in neoadjuvant treated primary breast-cancer and their correlation to pathological-response and overall survival.

**Methods:**

We analyzed immunohistochemical expression of Slug, Sox9 and Sox10 in tissue microarrays of 96 breast cancers prior to and after neoadjuvant chemotherapy. Expression was evaluated in invasive tumor cells and in tumor stroma and scored as 0, 1+, 2+ 3+. Expression-profile prior to and after chemotherapy was correlated to overall survival (Kaplan Meier) and with established clinico-pathological parameter.

**Results:**

Sox9, Sox10 and Slug were expressed in 82–96% of the tumor cells prior to chemotherapy. Slug was expressed in 97% of the cases in tumor stroma before therapy. Change in expression-profile after chemotherapy occurred only in Slug expression in tumor-cells (decreased from 82 to 51%, p = 0.0001, Fisher’s exact test). The other markers showed no significant change after chemotherapy. Stromal Sox9 expression (0 to 2+) correlated to better overall survival after chemotherapy (p = 0.004) and reached almost statistical significance prior to chemotherapy (p = 0.065). There was no correlation between Sox9 and hormone-receptor expression. In multivariate-analysis, the stromal Sox9 expression after chemotherapy proved to be an independent and better prognostic marker than hormone-receptor status. Other clinico-pathological parameter (as HER2-status or pathological-stage) showed no correlation to the analyzed markers.

**Conclusion:**

Strong stromal Sox9 expression in breast cancer after chemotherapy was found to bear negative prognostic information and was associated with shortened overall survival. Slug expression was significantly changed (reduced) in samples after neoadjuvant chemotherapy.

## Introduction

Prognosis for breast cancer has improved continuously during the last decades but it is still one of the most frequent causes of tumor related death in women in the western world. Possible reasons of breast cancer mortality are tumor dormancy after treatment followed by local, regional or distant recurrence. Several factors as hormone receptors, HER2 status, proliferation fraction predicting outcome in preoperative setting were extensively examined in previous studies (Chen et al. 2013; Denkert et al. 2010; Lips et al. 2013; Payne et al. 2008; Teleki et al. 2013; van Nes et al. 2012; Varga et al. 2005; Yoshioka et al. 2013). The role of transcription factors (TF) in breast cancer prognosis has been the subject of some previous studies (Ablett et al. 2012; Cimino-Mathews et al. 2013; Giordano et al. 2012; Guo et al. 2012; Mego et al. 2012). Slug (SNAI2), a transcriptional repressor, is member of the Snail family of zinc finger proteins and capable to act as a master regulator, altering expression of a number of genes including E-cadherin, a transmembrane protein which plays an important role in cell adhesion (Guo et al. 2012). The role of Slug in cancer developmental processes has been highlighted in several publications (Markiewicz et al. 2012; Mego et al. 2012; van Nes et al. 2012). It has been discovered that Slug is involved in an early developmental phenomenon known as 'Epithelial to Mesenchymal Transition’ or EMT, which results in the acquisition of an invasive, mesenchymal phenotype by epithelial cells. It has been postulated to play an important role in cancer growth and metastases spreading (Guo et al. 2012). Slug and Sox9 were shown to induce epithelial mesenchymal transition (EMT) and their expression consistently defines mammary stem cell state (Guo et al. 2012). Sox9 and Sox10 are two of the 20 different human Sox genes that also encode family of transcription factors (Chakravarty et al. 2011; Muller et al. 2010; Smalley et al. 2013; Soady & Smalley 2012). In cooperation with Slug, Sox9 can be used to determine the mammary stem cell state (Soady & Smalley 2012). Sox9, a nuclear TF, is often localized in cytoplasm of invasive and metastatic breast cancer (Chakravarty et al. 2011). It has been seen that patients with elevated Sox9 levels in cytoplasm suffer from faster tumor cell proliferation and significantly shorter overall survival (Chakravarty et al. 2011).

Sox10 was described last year to be present in invasive breast cancer especially in triple negative phenotype (Cimino-Mathews et al. 2013). Sox9, Sox10 and Slug were shown to be associated with poor overall survival in breast cancer (Cimino-Mathews et al. 2013; Guo et al. 2012).

In surgical pathology Sox10 - a TF that allows the survival and differentiation of neural crest cells into mature cells - is honored to support the diagnosis of melanoma and nerve sheath tumors (Mohamed et al. 2013; Shin et al. 2012). Recently Sox10 labeling has been documented in myoepithelial differentiated cells in salivary gland neoplasms and also in metaplastic triple negative breast cancers (Cimino-Mathews et al. 2013; Ivanov et al. 2013).

The aim of this study was to discover the prognostic role of Slug, Sox9 and Sox10 in neoadjuvantly treated breast cancer and the correlation to pathological response and overall survival. Furthermore, we tested stability of these markers during chemotherapy.

## Materials and methods

### Patient cohort

96 breast cancer patients, diagnosed by tissue core needle or fine needle aspiration biopsy (FNAB) and treated with preoperative neoadjuvant chemotherapy were selected consecutively between 1998 and 2009 out of the archives of the Institute of Surgical pathology, University Hospital Zürich, Switzerland. The treatment regimens encompassed different modalities including Herceptin combined with Taxol or 2 to 6 cycles of Fluorouracil- Epirubicin and/or Cyclophosphamid/Epi-docetaxel.

For 64 out of 96 patients, formalin-fixed, paraffin-embedded (FFPE) tumor blocks from preoperative core biopsies and corresponding postoperative operation specimens were available. Core biopsies prior to neoadjuvant chemotherapy without postoperative surgical specimens were available in 17 patients. For 15 patients, postoperative surgical specimens were available without previous core biopsies. Clinico-pathological data and follow up information (2-10 years) on all 96 patients could be retrieved from the pathological and clinical files.

75 tumors were histologically/cytologically diagnosed as an invasive ductal carcinoma (75/96: 78%), 18 cases as invasive lobular carcinoma (18/96: 19%), 2 more were categorized as metaplastic squamous cell carcinoma (2/96: 2%) and 1 case as a small cell carcinoma (1/96: 1%). The age of the patients was ranged between 30 and 74 years; mean age 52 years. Histological grading could be done in 81 cases using the modified Bloom and Richardson score: 38 of 96 carcinomas were poorly differentiated (grade 3), (40%), 42 cases were moderately differentiated (grade 2) (44%) and one case was well differentiated (grade 1) (1%). In 15 cases correct histological grading on the core biopsy was not possible due to the too small amount of tumor tissue.

Surgery was performed as follows: Mastectomy in 60 patients, segmentectomy, in 28 patients. Breast surgery was combined with axillary dissection in all but 5 of these patients. Residual tumor tissue after completing preoperative chemotherapy was determined as recommended in the residual cancer burden guidelines as the percentage of residual tumor cells distributed in the tumor bed area (Symmans et al. 2007).

Details of clinic-pathological parameter are shown in Table 1. Table with patient’s collective in more details previously published (Teleki et al. 2013).

**Table 1:** **Clinico-pathological parameter of the breast cancer samples in the tissue micro arrays**

N = 96	Prior to chemotherapy		After chemotherapy	
Tumor size	cT1	1 (1%)	ypT0	6 (6%)
	-a		ypT1	20 (21%)
	-b		-a	2
	-c	1	-b	11
	cT2	25 (26%)	-c	7
	cT3	22 (23%)	ypT2	31 (32%)
	cT4	41 (43%)	ypT3	24 (25%)
	-b	19	ypT4	7 (7%)
	-d	22	-b	6
	NA	7 (7%)	-d	1
			No surgery	8 (9%)
Nodal status	cN0	10 (11%)	pN0	25 (26%)
	cN1	62 (64%)	pN1	27 (28%)
	cN2		pN2	12 (12%)
	cN3	3 (3%)	pN3	14 (15%)
			no surgery	8 (9%)
	NA	21 (22%)	NA	10 (10%)
ER status	Positive	68 (71%)	Positive	59 (62%)
	Negative	25 (26%)	Negative	16 (17%)
	NA	3 (3%)	NA	21 (21%)
			(ypT0 or no surgery)	
PR status	Positive	59 (62%)	Positive	46 (48%)
	Negative	34 (35%)	Negative	29 (31%)
	NA	3 (3%)	NA	21 (21%)
			(ypT0 or no surgery)	
HER2 status	Positive	28 (29%)	Positive	18 (19%)
	Negative	65 (68%)	Negative	57 (60%)
	NA	3 (3%)	NA	21 (21%)
			ypT0, no surgery	

*Abbreviations:* *NA* not available, *ER* estrogen receptors, *PR* progesteron receptors.

The study and the construction of the TMA was approved by the Ethical Committee of the Canton Zürich (KEK- ZH NR: 2009-0065) and also by the Internal Review Board of the Institute of Surgical Pathology.

### Detection of hormone receptors (ER/PR) and HER2 status

Estrogen receptors (ER, clone 6F11) and progesterone receptor (PR, clone 1A6) expression was determined using the iVIEW DAB detection kit in Ventana Benchmark (all from Ventana, Basel, Switzerland) immunostainer following heat induced epitope retrieval in CC1 solution. According the current guidelines, at least 1% nuclear positive tumor cells were considered to be positive (Hammond et al. 2010).

HER2 status was defined according to the initial and the modified ASCO criteria using immunohistochemistry (IHC) and/or fluorescence in situ hybridization (FISH) (between 1998-2004 IHC complemented with FISH, between 2004-2009 FISH only methdology).

For immunohistochemistry, the CB11 clone of Anti- Her2 monoclonal antibody (Ventana) was used for automated immunostaining as mentioned above. Scoring was used in agreement with the time current FDA and ASCO/CAP guidelines (Lebeau et al. 2001; Wolff et al. 2007). Cases with tumor cells of > 10% strong and complete membrane staining were considered 3+, cases with moderate and complete membrane staining tumor cells were defined to be 2+. For FISH, the *HER2* gene amplification was tested using the dual color FISH kit of PathVision (Vysis, Abbott AG, Baar, Switzerland) following the manufacturer’s protocol. FISH reactions were evaluated using an Olympus computer guided fluorescence microscope (BX61, Olympus AG, Volketswil, Switzerland). Scoring was done following the time current FDA and ASCO/CUP guidelines: amplified status was diagnosed when ratio (between HER2 gene and chromosome 17) was >2.0 (until 2007) resp. >2.2 (from 2008).

### Tissue microarray construction

All cases were re-evaluated on hematoxylin-eosin (HE) stained sections of the FFPE tumors for suitability for the tissue microarray (TMA). Tumor tissues from 81 patients prior to chemotherapy and tumor samples from 79 patients after chemotherapy were arrayed into two TMA blocks using methodology described earlier (Kononen et al. 1998; Theurillat et al. 2007). Matched tissue samples before and after neoadjuvant chemotherapy were available for 64 patients. From every patient duplicated cores of tissue samples were arrayed into the cores.

### Immunohistochemistry detection Slug, Sox9 and Sox10

Slug, Sox9 and Sox10 were detected using immunohistochemistry on the fully automated Ventana Benchmark autostainer following the manufacturers’ instructions. Following antibodies was used: Slug (Cell Signaling Technology, C19G7, 1:100), Sox9 (Millipore, AB5535, 1:400), Sox10 (1:50, Santa Cruz Biotechnology, Santa Cruz CA). IHC stains for Slug and Sox9 were homogenous across entire tumor areas.

Expression for all three markers was evaluated in invasive tumor cells and in tumor stroma and scored as 0, 1+, 2+ 3+. Expression profile prior to and after chemotherapy was correlated to overall survival (Kaplan Meier) and with established clinico-pathological parameter.

### Statistical analyses

SPSS 15.0 software was used for statistical comparisons (SPSS, Inc., Chicago, IL, USA). Categorical data were analyzed using Chi-Square test. Spearman rank correlation was used to correlate Slug, Sox9, Sox10 expression and clinic-pathological parameters (stage and grade, hormone receptor status, Her2 status). Kaplan-Meier method was used to calculate overall survival with evaluation of statistical significance by log rank test. Multivariate analysis was performed using Cox regression method with 95% confidence intervals by including Sox9 and hormone receptor status both of before and after chemotherapy. These parameters did not correlate directly each other. Results were statistically significant at p values of < 0.05. Bonferroni correction was not applied.

## Results

### Sox9, Sox10 and Slug expression before and after neoadjuvant treatment and stability of expression profile during chemotherapy

Sox9: Before treatment 87% (73 of 84 cases) of the tumors cells were Sox9 positive. 23% (19 of 84 cases) showed expression of Sox9 in the stroma. After chemotherapy there were 88% (72 of 82 cases) tumors with Sox9 expression in the tumor cells and 21% (19 of 84 cases cases) with expression in the tumor stroma.

Sox10 was positive in 94% of the cases (79 of 94 cases) in the tumor cells before chemotherapy and 91% (75 of 82 cases) after treatment. There was no positivity in stroma with Sox10.

Sox9 and Sox10 showed no significant change in expression profile after treatment and remained stable stable during chemotherapy.

Slug was expressed in 82% of the tumor cells (69 of 84) and in 97% of the cases (81 of 84) in the tumor stroma prior to chemotherapy. Slug showed relevant changes after chemotherapy: 51% of the cases (42 of 82) were Slug positive in the tumor cells. 74 of 82 cases were positive for Slug in the stroma (90%). Strong (score 3) positivity in stroma decreased form 57% (48 of 84) to 3% (3 of 82).

**Figure 1:**
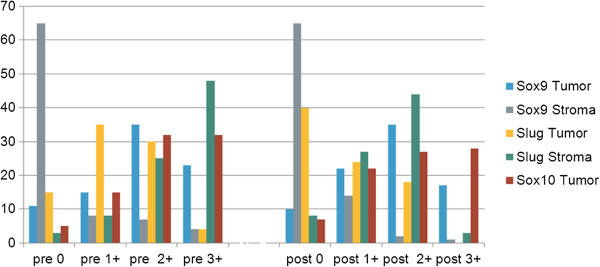
**The presence of Sox9, Sox10 and Slug in tumor and stroma was compared before and after treatment in our collectiv of breast cancer patients.** They are grouped in 4 categories of marker expression, to be specific: no expression = 0,low expression = 1+, moderate expression = 2+, high expression = 3+.

Details of scores of immunohistochemistry are shown in Table 2. and in in graphical illustration in Figure 1.

**Table 2:** **Distribution of marker expression in tumor and stroma, distinguishing between 4 different expression groups such as 0 = no expression, 1+ low expression, 2+ intermediate expression, 3+ strong expression**

n = 84	SOX9	SOX9	SLUG	SLUG	SOX10
(pre)	Tumor	Stroma	Tumor	Stroma	Tumor
**pre 0**	11	65	15	3	5
	(13%)	(77%)	(18%)	(3%)	(6%)
**pre 1+**	15	8	35	8	15
	(18%)	(10%)	(41%)	(10%)	(18%)
**pre 2+**	35	7	30	25	32
	(41%)	(8%)	(36%)	(30%)	(38%)
**pre 3+**	23	4	4	48	32
	(28%)	(5%)	(5%)	(57%)	(38%)
**n = 82**					
**(post)**					
**post 0**	10	65	40	8	7
	(12%)	(79%)	(49%)	(10%)	(55)
**post 1+**	22	14	24	27	22
	(26%)	(17%)	(29%)	(33%)	(26%)
**post 2+**	35	3	18	44	27
	(42%)	(3%)	(22%)	(54%)	(33%)
**post 3+**	17	1	0	3	28
	(20%)	(1%)	(0%)	(3%)	(34%)

**Figure 2:**
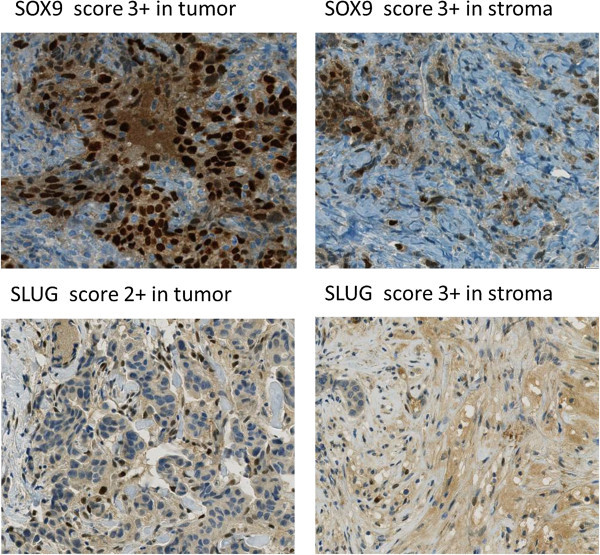
**Immunohistochemical expression of Sox9 and Slug in tissue microarrays showed in tumor compared to the expression in stroma.**

Examples of immunohistochemical stains are illustrated in Figure 2.

### Statistical analysis

#### Correlation of Slug, Sox9 and Sox10 expression to overall survival (OS)

Sox9, Sox10 and Slug prior to and after chemotherapy were correlated with overall survival (Kaplan-Meier). Only the stromal Sox9 expression prior to and after chemotherapy showed correlation with OS Cases with 0, 1+, 2+ expression of stromal Sox9 pre-chemotherapy had a nearly significant better overall survival than those of 3+ (p=0.065).

#### Correlation of Slug, Sox9 and Sox10 expression to ER/PR and HER2 status

ER and PR status prior to and after chemotherapy showed correlation with overall survival (pER-pre=0.025; pER-post=0.044; pPR-pre=0.045; pPR-post=0.067). ER and PR status did not show correlation with Sox9 (chi square). ER and PR expression showed positive correlation with each other (p=0.002). Hormone receptor status and stromal Sox9 prior to and after chemotherapy were involved separately in the multivariate analysis (Cox regression). HER2 status did not correlate with overall survival in this cohort, so it was ignored from multivariate analysis.

**Figure 3:**
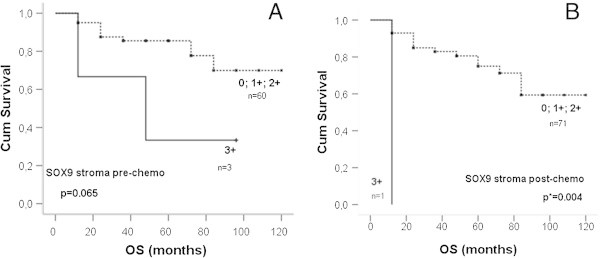
**Kaplan Meier curves in stromal SOX9 expression. A**: Cumulative survival in months of Sox9 expression prior to chemotherapy was compared to high expression (3+) und moderate/ no expression (0, 1+, 2+), log-rank P = 0.065. **B**: Post-chemotherapy survival in months, P = 0.004.

According to multivariate analysis, stromal Sox9 expression after chemotherapy proved to be an independent and better (but negative) prognostic marker than hormone receptor status. Prior to chemotherapy the prognostic power of stromal Sox9 is similar than the hormone receptor status (Table 3). Cases with 0, 1+, 2+ expression of stromal Sox9 post-chemotherapy had significantly better overall survival than those of 3+ (p=0.004) Figure 3.

**Table 3:** **Multivariate Cox regression analysis of hormone receptor status and stromal Sox9 expression before and after chemotherapy**

Parameters	P-value	HR	95% CI	
			Lower	Upper
ER_pre_	0.052	3.139	0.988	9.969
Sox9_pre_ stroma	0.063	0.234	0.050	1.081
PR_pre_	0.015	0.227	0.069	0.746
Sox9_pre_ stroma	0.029	0.169	0.034	0.834
ER_post_	0.077	2.796	0.893	8.753
Sox9_post_ stroma	0.006	0.037	0.004	0.384
PR_post_	0.223	2.062	0.644	6.609
Sox9_post_ stroma	0.034	0.082	0.008	0.833

pre: before chemotherapy; post: after chemotherapy.

HR: hazard ratio; CI: confidence intervals.

#### Change in Slug, Sox9 and Sox10 expression profile prior to and after chemotherapy

Slug stromal expression (score 3) showed highly significant changes after chemotherapy (dropped from 57% to 3%), p = 0.0001 (Fishers’ exact test).

Lack of Slug expression in the tumor cells became more frequent after chemotherapy and proved also significant (increased from 18% to 49%), p = 0.0029 (Fishers’ exact test).

There was no relevant or significant change in the expression profile of Sox9 and Sox10 after chemotherapy.

## Discussion

In our study we analyzed the prognostic role and stability of transcription factors as Sox9, Sox10 and Slug in a cohort of preoperative chemotherapeutically treated breast cancers. We could show in this cohort that strong stromal Slug expression was instable during chemotherapy and postoperative stromal Sox9 expression was significantly associated with shortened overall survival.

We are not aware of any studies in the literature, showing association between these transcription factors and prognosis in a preoperative treated breast cancer cohort. In the last two years different studies addressed to the Sox genes (Chakravarty et al. 2011; Muller et al. 2010; Soady & Smalley 2012). It was demonstrated that these genes play an important role in cancer development as well. Differentiated mammary luminal cells expressing both Sox9 and Slug, an EMT-associated transcription factor, were able to activate an endogenous autoregulatory network in adult stem cells and reconstitute an entire mammary gland (Guo et al. 2012). Meanwhile, showing stem cell characteristics such as self-renewal and multi- potency, Sox10 is one of the most studied Sox family genes, mainly because of their role in the survival of neural crest cells and compilation into neural-crest-derived melanocytes and glia (Cimino-Mathews et al. 2013; Ivanov et al. 2013). Invasive breast carcinomas, especially those with triple negative hormone expression and metastases seem to be supportive for myoepithelial differentiation (Cimino-Mathews et al. 2013; Ivanov et al. 2013). Expression of Sox9, Sox10 and Slug was seen in 82-96% of the tumor cells prior to chemotherapy in our study, further supporting the fact that the transcription factors are highly active in the breast cancer tissue.

It was described in a recent work of A. Cimino-Mathews et al., that Sox10 is also expressed in breast carcinomas, mainly in triple negative cases, changing the previous view on Sox10 being exclusively expressed by melanocytic lesions (Cimino-Mathews et al. 2013).

Our cohort is a mainly hormone receptor dominated tumor collection. Interestingly, Sox10 was detected in a high percentage of tumor samples (up to 91-94%). The high expression of Sox10 might represent a rather unspecific staining property of unknown biological significance as staining intensity did not have any prognostic impact in this patients’ cohort. Moreover, Sox10 remained stable during chemotherapy showing neither correlation to overall survival nor to response to chemotherapy. This is most likely due to the fact that hormone receptor positive carcinomas of the breast, as was the case in our study, can benefit from different therapeutic options than triple negative or basal like cancers. Further prognostic role of Sox10 in breast cancer, needs to be addressed in future studies.

Co-expression of Slug and Sox9 in breast cancer was found to correlate with rapid tumor growth and metastasis spreading in a study earlier (Guo et al. 2012). In other studies, the high expression of Sox9 was found to be associated with higher histological grade and/or triple negative phenotype and thus considered as prognostic marker in adjuvant oncological settings (Chakravarty et al. 2011). In our cohort of neoadjuvantly treated breast cancers, strong Sox9 expression (score 3) in tumor stroma significantly correlated with shortened overall survival after completion of preoperative chemotherapy, further supporting the hypothesis described by Guo et al (Guo et al. 2012). The lack of correlation between weaker Sox9 scores and survival can be most likely explained by the fact that our cohort included mainly hormone receptor positive cases. Strong stromal Sox9 expression may be potentially used as a prognostic marker additionally to conventional prognostic parameter when assessing residual tumor burden and response after chemotherapy.

As to expression of Slug, we could show a drastic drop in the stromal Slug (score 3) expression after chemotherapy. Furthermore, we detected significantly more tumors lacking Slug expression in the tumor cell postoperatively. As Slug expression could not be correlated to any known prognostic factors in our cohort, the biological relevance of this phenomenon remains unclear at the moment.

The correlation of Sox9, Sox10 and Slug to other classical clinico-pathological parameters as hormone receptors, HER2 and proliferation index could confirm that Sox9 is an independent negative prognostic factor. This finding may be used as additional information on overall survival and therapy response in neoadjuvant treatment setting.

In summary, our study shows, that strong stromal SOX9 expression in breast cancer after neoadjuvant chemotherapy bears negative prognostic information and is associated with shortened overall survival. The biological relevance of change in phenotype change resp. in expression profile of Slug, both in stroma and tumor cells, needs to be addressed in further studies.
